# A two-year retrospective statistical analysis of trauma cases in our institute

**DOI:** 10.1186/1865-1380-7-S1-P4

**Published:** 2014-07-25

**Authors:** Sateesh Kumar Muppasani, Vijaya Durga Reddy

**Affiliations:** 1 S.V.S. Medical College, Venugonda, Mahabubnagar, Andhra Pradesh, India

## Objective

This is a retrospective study of trauma cases from January 2007-December 2009 (212 patients) to determine the incidence of gender, age, alcohol influence, mode of injury, time of injury, nature of injuries, hospital stay and to suggest preventive methods.

## Methods

This is a retrospective study of trauma cases from January 2007-December 2009 (212 patients). After obtaining permission from the concerned authorities, a pre designed questionnaire was used to collect data from emergency department. the data being computerised and analysed.

## Results

Road traffic accidents (RTA) were the common cause of morbidity and mortality in India. Among 212 trauma patients 79.70% were road traffic accidents, men sustained more injuries (84%), mostly in the age group of 20-30 yrs (73%). Most of them sustained fractures (50.47%), and most of the accidents occurred during night time (58%), afternoon (45%), evening (44%).

## Limitations

The duration of the study was only two years, no data was available regarding out of hospital trauma, and road safety methods they have taken and the cases could not be followed up post operatively. The need for prospective studies to further support and validate the findings of the study.

## Conclusion

Road traffic accidents are the most common cause of morbidity and mortality in young males, mostly occurring during night, and most of them sustained long bone fractures.

To minimise road traffic accident incidents avoid nighttime travelling.

